# Optical Fiber Array Sensor for Force Estimation and Localization in TAVI Procedure: Design, Modeling, Analysis and Validation

**DOI:** 10.3390/s21165377

**Published:** 2021-08-09

**Authors:** Naghmeh Bandari, Javad Dargahi, Muthukumaran Packirisamy

**Affiliations:** 1 Robotic Surgery Laboratory, Mechanical, Industrial, and Aerospace Engineering Department, Concordia University, Montreal, QC H3G 2W1, Canada; nag_moha@encs.concordia.ca (N.B.); dargahi@encs.concordia.ca (J.D.); 2 Optical Bio-Micro Systems Laboratory, Mechanical, Industrial, and Aerospace Engineering Department, Concordia University, Montreal, QC H3G 2W1, Canada

**Keywords:** optical sensor, transaortic valve implantation, force, sensing, localization, finite element method

## Abstract

Transcatheter aortic valve implantation has shown superior clinical outcomes compared to open aortic valve replacement surgery. The loss of the natural sense of touch, inherited from its minimally invasive nature, could lead to misplacement of the valve in the aortic annulus. In this study, a cylindrical optical fiber sensor is proposed to be integrated with valve delivery catheters. The proposed sensor works based on intensity modulation principle and is capable of measuring and localizing lateral force. The proposed sensor was constituted of an array of optical fibers embedded on a rigid substrate and covered by a flexible shell. The optical fibers were modeled as Euler–Bernoulli beams with both-end fixed boundary conditions. To study the sensing principle, a parametric finite element model of the sensor with lateral point loads was developed and the deflection of the optical fibers, as the determinant of light intensity modulation was analyzed. Moreover, the sensor was fabricated, and a set of experiments were performed to study the performance of the sensor in lateral force measurement and localization. The results showed that the transmitted light intensity decreased up to 24% for an external force of 1 N. Additionally, the results showed the same trend between the simulation predictions and experimental results. The proposed sensor was sensitive to the magnitude and position of the external force which shows its capability for lateral force measurement and localization.

## 1. Introduction

Transcatheter procedures are among the most expanded areas in minimally invasive surgeries (MISs). During such procedures, catheter-based techniques (CBTs) are used to perform surgery or intervention on the heart and vasculature. A catheter is a thin flexible tube that is inserted into the patient’s blood vessels and advanced towards an intraluminal anatomic site, e.g., coronary arteries, via small incisions in the femoral or radial artery or veins. Such procedures are performed for both diagnosis and treatment purposes [1]. Simultaneously, surgeons use X-ray fluoroscopy for real-time imaging and navigation of the catheter and surgical instruments. CBT procedures may also be performed under magnetic resonance (MR) imaging for navigation purposes [2], especially in neurovascular surgery. A CBT is more favorable clinically in comparison with conventional open surgery. It has a shorter time of surgery and less anesthesia agent usage and surgical trauma. In addition, blood loss, pain, tissue scarring, hospitalization and recovery time are effectively reduced with CBTs [3].

During the last decade, the CBT approach was adopted for cardiac valve replacement. Heart valve anomalies, e.g., aortic stenosis (AS), are among the most prevalent cardiac diseases in the senior population. Conventionally, AS has been treated through open-heart aortic valve replacement (AVR). Open AVR is associated with a mortality rate of up to 7% [4]. As an alternative, transcatheter aortic valve implantation (TAVI) has gained adoption as an MIS alternative to AVR, especially for the elderly or patients with a co-morbidity who may not tolerate long anesthesia times. TAVI technology has shown superior clinical outcomes compared to open AVR. However, it has limitations in terms of accurate placement of the valve within the heart. Constrained maneuverability of the valve during deployment, 2D projection and poor soft tissue contrast in X-ray fluoroscopy images and a lack of tactile feedback affect the surgeon’s perception of the position and the orientation of the valve, which often leads to misplacement of the valve. Inaccurate deployment of the artificial aortic valve could lead to regurgitation, stroke, insufficient coronary perfusion, cardiac blockage and death [5]. The commercially available TAVI solutions, e.g., CoreValve (Medtronic Corp., Dublin, Ireland), and Sapien (Edwards Lifescience Corp., Irvine, CA, USA), typically have a 16–18 Fr diameter (1 Fr = ⅓ mm and is pronounced as ‘French’). However, there is no tactile sensor to assist surgeons in localization of the aortic annulus to deploy the prosthetic valve.

More specifically for TAVI and despite its advantages over AVR, its main technical limitation is the loss of natural tactile sensing. Especially in robot-assisted surgeries, e.g., with the system proposed in [6], the loss of tactile feedback is shown to adversely affect the ability of surgeons in estimation of tool–tissue interaction force or tissue distinction [7,8]. Consequently, surgeons are less situationally aware of the surgical site during TAVI procedures, which may result in risky surgical maneuvers and irreversible or catastrophic trauma to the patient. To provide tactile feedback during CBT procedures, researchers have investigated different tactile sensors to be mounted on CBT instruments and compensate for the lost tactile feedback [8,9,10,11,12,13,14,15]. Such a tactile sensor shall be small enough to fit at the tip of these catheters, typically of 2–6 mm in diameter. In addition, these sensors should be biocompatible, electrically passive and MRI compatible to be safe in the operation room and inside the patient’s body [4,16]. Additionally, these sensors should work in both static and dynamic loading conditions while showing low hysteresis and non-linearity [17]. The output of such tactile sensors can be relayed to surgeons via visual feedback [18,19,20] or through tactile displays [21,22,23,24]. The choice of the sensing principle for tactile sensors highly depends on the requirements of the surgical application. While the mentioned requirements are common, a complete set of functional and physical requirements for such tactile sensors is application dependent and some requirements may vary slightly among various surgical procedures [25]. The authors have reported a comprehensive review on functional and physical requirements of tactile sensors for different MIS procedures in [9].

Among various sensing principles, e.g., electrical sensing and optical sensing, optical-based sensing principles exhibit the most compatibility with the MIS requirement [9]. Optical-based tactile sensors are mainly based on wavelength modulation, phase modulation and light intensity modulation (LIM) [9]. The main component used in such tactile sensors’ structure is an optical fiber.

In recent years, various optical fiber sensors have been proposed to measure force, displacement, pressure, stiffness and temperature to compensate for the loss of tactile feedback in MIS [9]. For example, Polygerinos et al. [26] proposed a LIM-based force sensor for cardiac catheters. This sensor was suitable for cardiac catheterization to detect the catheter tip interaction force with the cardiac wall. The sensor had a 3 mm diameter and could measure contact force in the range of 0–0.85 N. In another study, Polygerinos et al. [2] proposed a triaxial catheter tip force sensor for the MRI-guided cardiac ablation procedure. They utilized a flexible structure with an integrated reflective surface placed in front of the optical fibers. Applying the force on the catheter tip, the distance and orientation of the reflector were changed and modulated the light intensity. The sensor showed high sensitivity and a working range of 0.5 N along all three orthogonal axes. Aditionally, Noh et al. [27] designed and proposed a small three-axis force sensor to measure the contact force during the cardiac ablation procedure. They coupled an optical fiber with a CCD camera and utilized an image processing technique to correlate the reflective intensity with external force on their sensor. However, their proposed multiple linear regression calibration had a noticeable error in comparison with the reference forces.

In another effort, Li et al. [28] proposed a novel 3D catheter distal force sensor for cardiac ablation using the wavelength modulation principle. They used fiber Bragg grating (FBG) in a force-sensitive flexure. They modeled three-axis force components and temperature using the decoupling principle. A 3D printing technique with non-magnetic material made the sensor capable of working in both X-ray- and MIR-guided cardiac ablation procedures. The sensor performance analysis showed linearity, repeatability and hysteresis errors of less than 5%. Recently, Li et al. [29] developed a triaxial FBG-based force sensor for enhanced force measurement in cardiac ablation procedures. The structure of their sensor was 3D printed with a force-sensitive fixture and consisted of five FBGs. Upon application of external force on the catheter tip, the strain-induced wavelength shift of FBGs was captured to calculate three components of the force. Additionally, to compensate for the thermal effect, they tested the sensor within a range of 25–50°, with 5° intervals and showed below 6.5% temperature-induced error. The range of measured force was −0.8 to 0.8 N in the transversal plane of the catheter and 0–0.8 in its longitudinal direction with an average error of 23.28 mN (2.91%). Ex vivo validation tests showed the performance and robustness of their sensor for detecting the tip contact force.

**Figure 1:**
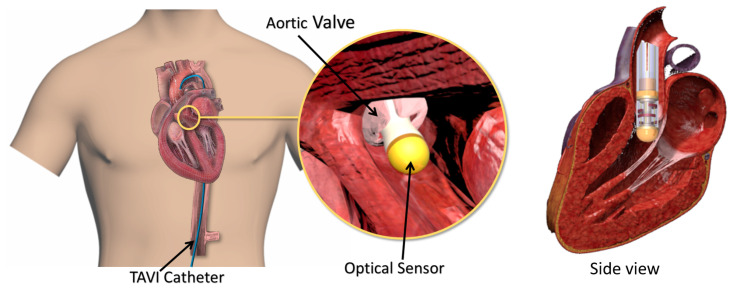
Schematic use case of the proposed sensor for aortic valve localization (the figure is not to scale).

Our literature review on sensorized catheters [9] showed that most of the tactile sensors proposed in the literature are intended for cardiac ablation procedures and are merely capable of measuring tip forces. Nevertheless, in cardiovascular CBT procedures, especially during TAVI, both the tip and side of the distal portion of the catheter are in contact with cardiac tissue. Moreover, to localize the aortic annulus, the sensor should be able to measure the lateral contact forces. The reason is that, as shown in Figure 1, the leaflets of the aortic valve (connected to the aortic annulus) will press on the body of the sensor from the lateral direction. Given that, during the pulsation, the leaflets open and close, a dynamic lateral contact force would be generated between the sensor and the leaflets. Therefore, the sensor must be capable of measuring the lateral forces and localizing the forces to localize the leaflets (and annulus).

The motivation for this study was to propose an optical fiber-based sensor capable of measuring and localizing the lateral contact forces for integration with a typical 18Fr TAVI catheter, also known as a delivery catheter. The proposed sensor is capable of measuring and localizing lateral contact forces. For clarity, measurement of contact point forces (at the tip) is out of the scope of this study. Thus, in this study, a conceptual optical fiber sensor based on bending-based intensity modulation was proposed, simulated and verified. The preliminary study of the conceptual design of the proposed sensor was first presented in [30]. The novelty of this work was in the ability of the sensor to measure the lateral contact forces between the valve leaflets connected to the annulus and the surrounding area of the sensor. This made the sensor capable of being used in TAVI procedures to locate the position of the annulus with respect to the sensor geometry. Additionally, an optomechanical model was developed based on the optical fiber bending loss and deflection of the optical fiber as an Euler–Bernoulli beam. The simulation was performed using finite element analysis, which predicted the sensor behavior with various forces applied longitudinally and circumferentially. The results of the simulation were verified in an experimental study aiming at showing the feasibility of the proposed sensor. In the following, Section 2 describes the sensor design requirements and the proposed structural design specifications. Optomechanical modeling, sensing principle and simulation are described in Section 3. Section 4 summarizes the sensor fabrication, test setup and validation protocol followed by results and a discussion in Section 5. Finally, the concluding remarks are provided in Section 6.

## 2. Design Requirements and Structural Design

As the sensor was intended for TAVI applications for annulus localization, it should have the ability to measure the bending-based light intensity modulation in the optical fibers caused by lateral contact forces between the sensor and aortic valve leaflets. Additionally, the sensor should be able to find the relative position of the annulus with respect to the sensor through localizing the contact forces. Moreover, the sensor should be capable of working under both dynamic and static conditions. In addition, the sensor should be small enough to be integrable at the tip of TAVI catheter and to move through vasculatures. Finally, the sensor needs to be biocompatible, electrically passive and MRI compatible.

**Figure 2:**
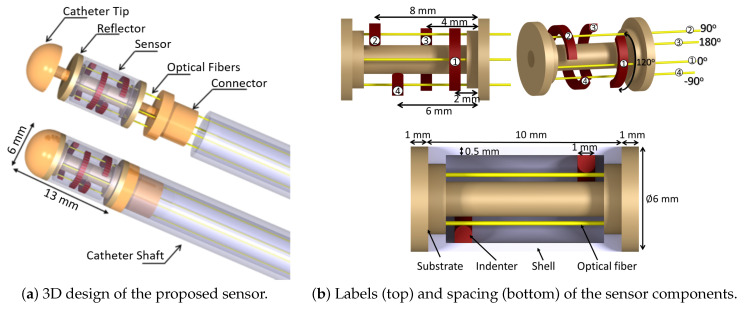
(**a**) Conceptual integration of the proposed sensor on a TAVI catheter, (**b**) cross-sectional view of the proposed sensor with the view of the flexible shell, indenters, substrate and the optical fibers.Cylindrical sensor for cardiovascular MIS applications.

Figure 2a depicts the proposed sensor. To accommodate the size constraint, the outer diameter of the sensor was considered to be 6 mm. The sensor was cylindrical and was covered with a flexible shell on the outer circumference. The shell had four internal indenters to transfer external force from the shell to the optical fibers. The indenters were distributed at 20% increments along the length of the shell and were consecutively separated by 90° in the circumferential direction. Each indenter was of semi-circular shape with a radius of 0.5 mm and circumferential span of 120°. The flexible shell was mounted on a rigid substrate which provided a base for the mounting of optical fibers. Four optical fibers passed through the proximal end of the substrate and finished at its distal end. The distal end of the substrate could be internally coated by gold or silver, e.g., through the sputtering technique, to reflect the light back into each fiber for a retro-reflection configuration. The cross-sectional view of the proposed sensor is shown in Figure 2b.

With this design, upon application of a point (or distributed) contact force on the outer shell, contact forces were transmitted in different proportions to the optical fibers and consequently caused light intensity modulation in each fiber. With the proposed design, each optical fiber acted as an Euler–Bernoulli beam with a circular cross-section fixed at both ends. The details of the geometric dimensions are provided in Table 1.

## 3. Sensor Modeling

In this section, the details of the sensing principle formulation and finite element simulation of the proposed sensor are provided.

### 3.1. Sensing Principle

The use case of the proposed sensor is in TAVI procedures. When the sensor is moved through the aortic valve (Figure 2b), the aortic leaflets open in the systolic phase and close in the diastolic phase of the heart cycle, thus applying pulsatile contact forces perpendicular to the flexible shell. The pulsatile forces are transferred to the optical fibers and cause bending of the fibers. The relatively long hanging span of the optical fibers and their relatively small flexural rigidity (EI) make them quite sensitive to small contact forces from the indenters.

**Figure 3:**
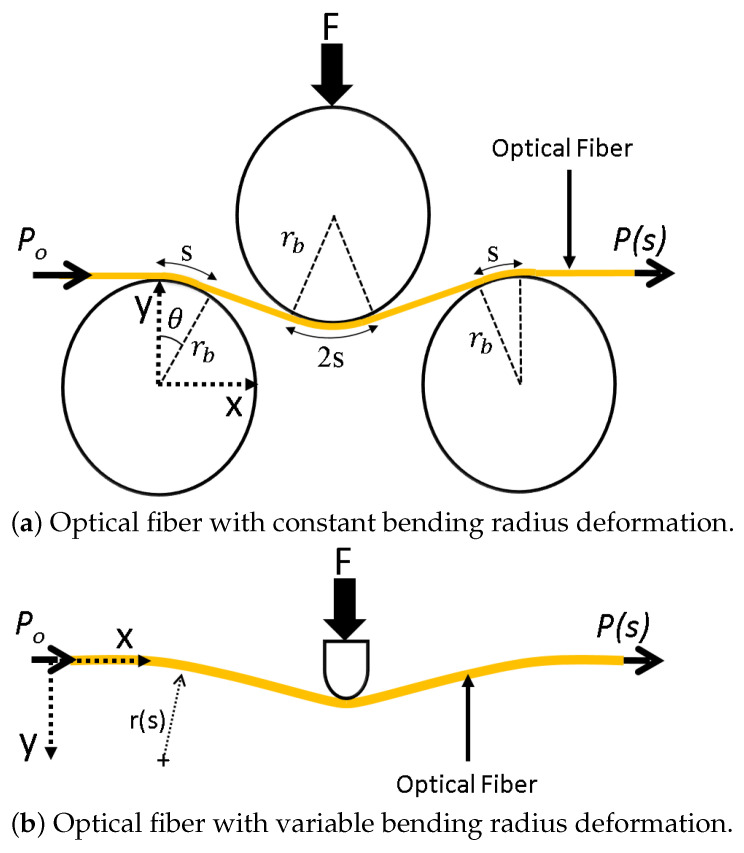
(**a**) Schematic of the constant bending radius principle, (**b**) schematic of the variable bending radius principle.

According to Gauthier [32], Equations (1) and (2) express the relationship between the bending-based power loss in an optical fiber with a constant radius of curvature:

(1) $$P\left(s\right)=P_{0}e^{-\gamma s},$$

(2) $$s=r_{b}\theta ,$$

where $P_{0}$ is the input power to the fiber, γ is an intrinsic optical constant of the fiber, $r_{b}$ is the radius of circular indenters, *s* is the arc-length parameter along the fiber, θ is the central bending angle and P(s) is the output power from the fiber. Figure 3a shows the schematic of the constant bending radius principle. As the equations indicate, the output power (and intensity) from a bent optical fiber, with a constant bending radius, depends on the total arc length, optical properties of the fiber and the bending angle.

In the case an optical fiber is bent as an Euler–Bernoulli beam, the radius of curvature is not constant and varies with arc length *s*. To accommodate that, the optical fiber was discretized into infinitesimally small elements which were assumed to be of constant bending radius. Using a differential form of Equation (1), and integration along *s*, the total bending-based power loss can be obtained. The authors have previously reported the derivation of this principle, variable bending radius (VBR), verification and validation in [31,33,34]. Figure 3b illustrates the variable bending radius principle.

Details of this derivation are provided in Section 3.1.

**Figure 4:**
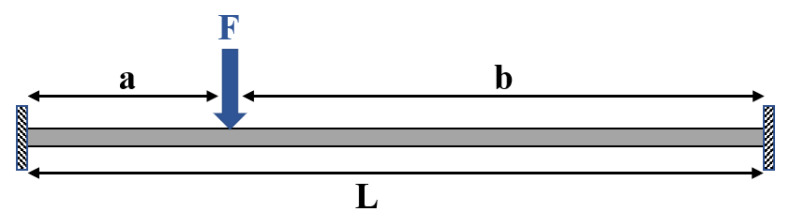
Simplified mechanical model of optical fibers in the proposed sensor with force *F* applied on it from the corresponding indenter.

To perform the integration, the existence of an analytical description of *r* and θ, as functions of *s*, is necessary. To obtain r(s) and θ(s), the infinitesimal strain form of Euler–Bernoulli theory was used. Each fiber was modeled as a finite strain Euler–Bernoulli beam, as depicted in Figure 4.

Equation (3) is the governing equation of the Euler–Bernoulli beam subjected to an internal moment of M(s) due to a lateral force *F* at a distance *a* from the left support. M(s) for such a configuration is:

(3) $$M\left(s\right)=\frac{EI}{r(s)}=\{\begin{array}{cc} -\frac{ab^{2}}{L^{2}}F+P_{1}s & 0<s\leq a\\ -P_{1}\left(s-a\right)-\frac{ab^{2}}{L^{2}}F+P_{1}s & a<s\leq L \end{array},$$

(4) $$P_{1}=\frac{b^{2}}{L^{3}}\left(3a+b\right)F,$$

(5) $$r\left(s\right)=\frac{{\left(1+{y^{\prime }}^{2}\right)}^{3/2}}{y^{\prime \prime }}\;\Rightarrow \;\mathrm{with}\;y^{\prime }\ll 1\;r\left(s\right)\approx \frac{1}{y^{\prime \prime }}$$

where ( )${}^{\prime }=\frac{d}{ds}$ is a derivative operator, $P_{1}$ is the reaction force at the left support and *y* is the deflection of the beam. As provided in Section 3.1, the substitution of Equations (3)–(5) in the differential form of Equations (1) and (2) and integrating over the length of the optical fiber provides an analytical relationship relating the external force *F* to the output power *P*.

### 3.2. Finite Element Simulation

**Figure 5:**
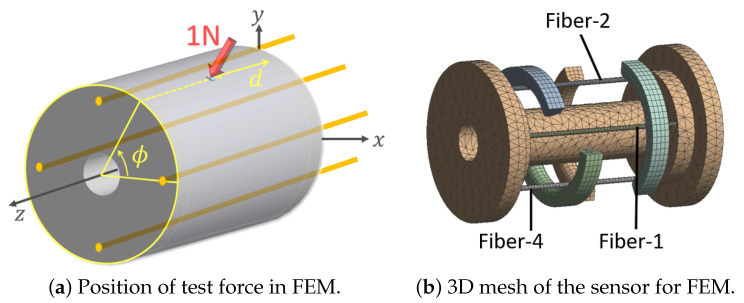
(**a**) Configuration of the external force on the flexible shell, (**b**) internal view of the meshed model used in the simulation.

To calculate the deflection of the shell and fibers with respect to the applied force, a three-dimensional finite element model of the sensor was developed in ANSYS Workbench v.16 (Ansys Inc., Canonsburg, PA, USA). Geometrical dimensions and material properties of the sensor’s structural components were assumed, as provided in Table 1. Since 3D printed materials were used for sensor fabrication, the material properties of the components were characterized using the authors’ characterization findings in [31]. The contact between each fiber and its corresponding indenter was assumed as frictionless for the sake of simplicity. The embedded ends of each fiber in the substrate (1 mm at each end) were fixed (no rotation, and no displacement). Additionally, both end surfaces of the substrate were fixed. An external static force of 1 N magnitude was applied on the flexible shell. To facilitate the numerical stability and to avoid stress singularity (to the machine precision) the external force was applied on a rigid block (0.1 × 0.1 mm) attached to the external surface of the flexible shell. A total of 72 simulations were performed for various combinations of longitudinal (4 *d*-s) and circumferential positions (18 ϕ-s) of the external force (and rigid block). Figure 5 shows the parametrized model and the meshed model with indenters. Table 2 shows the simulated geometric parameters and their values. The geometric model was meshed with quadratic brick and tetrahedron elements (n≈27,000). The solutions were performed using the static solver with a large deformation effect enabled.

**Figure 6:**
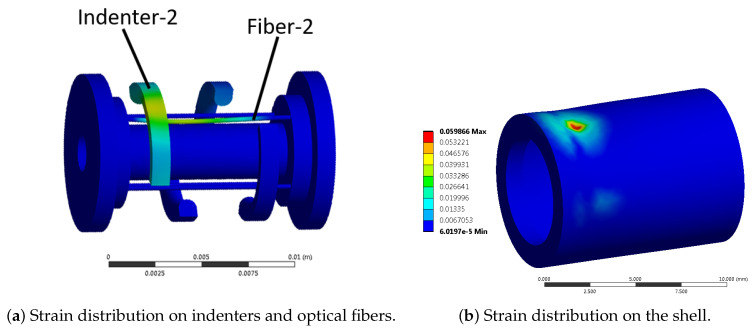
Distribution of von Mises strain for load case (d=8,ϕ=90) in: (**a**) Fiber-2 and Indenter-2, and (**b**) the flexible shell.

The finite element model was solved for all possible combinations of parameters, i.e., (*d*, ϕ)-s. Figure 6 depicts the distribution of von Mises strain within the indenters, fibers and membrane for a representative configuration when the external force was applied exactly on top of Indenter-2. The maximum von Mises strain of the shell in the depicted simulation was approximately 0.06 which is beyond the finite strain limit of 0.002, thus confirming the assumption of finite strain.

Table 3 shows the maximum deflection of fibers in simulations. In all cases, the maximum deflection in each fiber was observed when the force was applied exactly on top of its corresponding indenter. The maximum deflection was observed in Fiber-3 and Fiber-4 with a value of 0.136 mm. The deflection to the free-length ratio for Fiber-3 and -4 at this deflection was $\frac{0.136}{8}=0.017$. Since this ratio is less than 0.05, the typical linear limit threshold for an Euler–Bernoulli beam [35], using the approximate definition of the radius of curvature (Equation (5)) in the sensing principle formulation, was justified.

**Figure 7:**
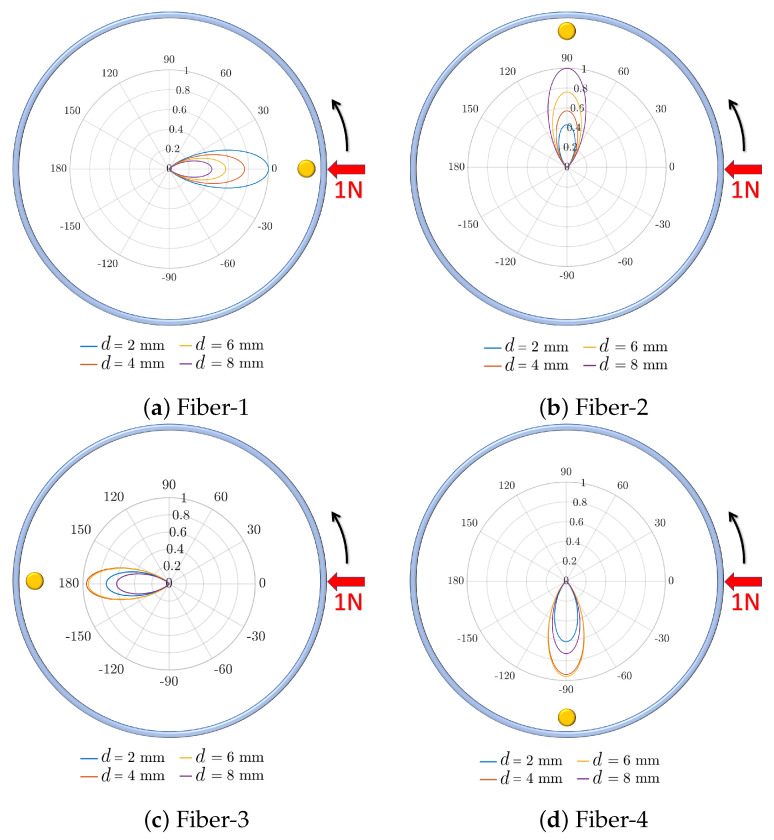
Normalized variation of the maximum deflection in (**a**) optical Fiber-1, (**b**) Fiber-2, (**c**) Fiber-3 and (**d**) Fiber-4 with respect to the change in the longitudinal and circumferential positions of external force.

Another result of the simulations was the variation of the maximum deflection of the optical fibers with respect to the circumferential location of the external force. The maximum deflection of the optical fiber was used as an indicator of the intensity loss in each fiber. Figure 7 depicts the polar diagram of the normalized maximum deflection of each fiber. This figure shows the maximum deflection of each fiber with respect to the circumferential position of external force and for four different longitudinal positions of the external force. The results showed that each fiber underwent its maximum deflection while the external force was applied on top of its corresponding indenter. Additionally, in positions where the force was out of the circumferential span of each indenter, its contacting fiber was almost shielded from external force by the membrane. However, the 120° indenter span would provide a 30° overlap between two consecutive indenters which would help in transferring force to two optical fibers when the external force is applied at a ϕ between two fibers.

## 4. Validation Study

In order to validate the proposed sensor concept, a series of experiments were performed. To this end, initially, a prototype of the sensor was fabricated. After that, components of the sensor and optical fibers were assembled. The image-based intensity DAQ system which was developed and validated previously in [36,37] was utilized in this study.

### 4.1. Sensor Fabrication

**Figure 8:**
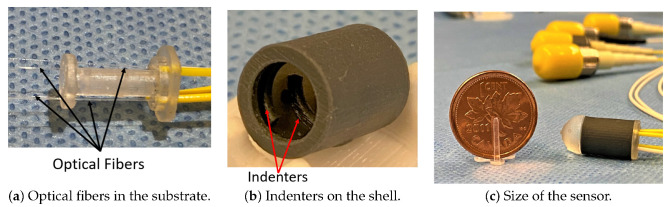
(**a**) A 3D printed rigid substrate and four inserted optical fibers, (**b**) 3D printed shell and indenters on its bottom surface using Formlabs flexible resin, (**c**) assembled structure of the cylindrical sensor in comparison with a coin, the cap is attached to show the final shape of the sensor with reflective surface suitable for TAVI procedures.Fabricated cylindrical optical-fiber array sensor.

The components of the sensor were fabricated with 3D printing technology using a Form2 3D printer (Formlabs Inc., Somerville, MA, USA). Formlabs clear resin was used to fabricate the sensor substrate. Additionally, for the flexible shell, Formlabs flexible resin was used. The indenters were printed at the bottom surface of the shell. This technique resulted in high spatial accuracy for indenter placement on the shell and eliminated the need for separate attachment and gluing. Figure 8 illustrates the sensor components and its assembled structure. The substrate facilitated passing four optical fibers with 90° angular separations, while each fiber was in contact with its corresponding indenter. However, since this study was a feasibility study and to reduce the complexity of data acquisition, only a single fiber (Fiber-1) was assembled with the prototyped sensor for validation testing. Nevertheless, the assembly of four independent optical fibers is shown in Figure 8 to represent the final assembly of the proposed sensor in practice.

### 4.2. Experimental Setup and Validation Test

**Figure 9:**
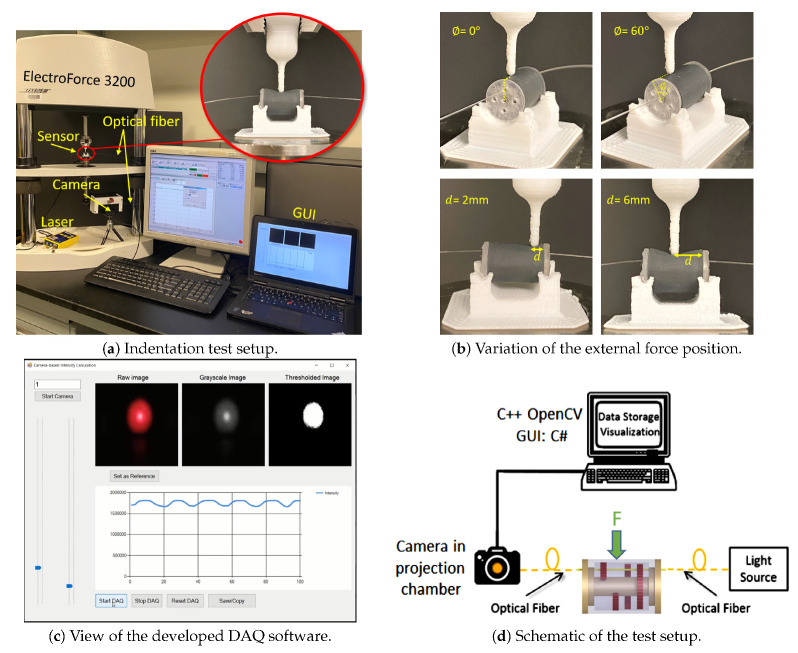
(**a**) Experimental setup, image-based DAQ and fabricated sensor under the test, (**b**) the prototype sensor under various load cases in the experimental study, (**c**) a representative view of the developed GUI software to record the intensity change in real time, (**d**) schematic system configuration.Experimental setup, image-based DAQ, and fabricated sensor under the test.

Figure 9a shows the experimental setup used for the sensor validation study. A laser source (OZ Optics, ON, Canada) supplied a 635 nm laser beam at a power of 5 mW to a coated single mode 250 μm diameter optical fiber (S405-XP, Thorlabs, NJ) with cladding and core diameters of 125 μm and 3 μm, respectively. Since the sensor had a symmetric design and fibers were fixed in similar configurations, only one optical fiber was inserted into the fabricated sensor. As illustrated in Figure 9a, the optical fiber was connected to an FC-PC connector attached to the projection chamber of the camera, a Logitech C920 (Logitech Inc., Lausanne, Switzerland)). Using the camera and image-based DAQ system would allow for using all the four fibers in future (with proper light isolation between the projected beams in the chamber).

For applying an external force, a universal testing machine (UTM) (ElectroForce^®^ 3200, TA Instruments, New Castle, DE, USA) was used. The sensor was located on a custom-designed holder which was adhered on the lower jaw of the UTM. A long thin indenter was installed on the upper and vertically movable fixture of the UTM to apply force on the sensor. For comparison, 1 N force was applied at a similar location as was considered in the finite element simulation. Meanwhile, the force was recorded through the UTM’s interface software and light intensity (passed through Fiber-1) was captured with the developed light intensity acquisition system. More specifically, the external force was a compressive sinusoidal force with a range of 0-1 N and frequencies of 0.5, 1.0 and 1.5 Hz. Such frequencies correspond to the heart’s beats per minute (BPM) of 30, 60 and 90. The external force was applied on the sensor at longitudinal locations d=(2, 4, 6, 8 mm) and at circumferential angles of ϕ= (−60°, −40°, −20°, 0°, 20°, 40°, 60°) for each *d*.

Since only one optical fiber, i.e., Fiber-1, was studied in the experiments, the tests were done on circumferential span and length associated with Indenter-1. Four representative images of the sensor under the test are provided in Figure 9b. Additionally, Figure 9c shows the GUI of the image-based intensity acquisition graphical user interface developed in C# language. In Figure 9c, three images of the laser spotlight are visible. The original image of the spotlight projected into the projection chamber (left), the grayscale converted image of the original image (center) and the binary mask showing the area of the spotlight are shown. The grayscale values of the pixels and the area of the laser spotlight were used to estimate the output light intensity using the method described in [36,37].

## 5. Results and Discussion

**Figure 10:**
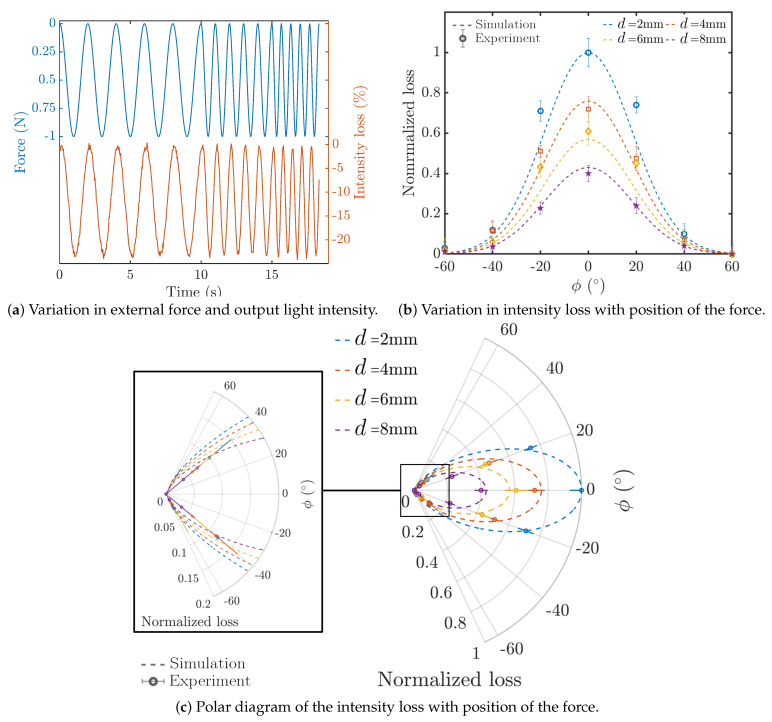
(**a**) Sensor response to 1 N concentrated force with frequencies 0.5, 1.0 and 1.5 Hz applied to the point (2 mm, 0°), and (**b**) comparison of the trends of change in the intensity loss of Fiber-1 with 1 N external force at various positions (Cartesian diagram), (**c**) comparison of the trends of change in the intensity loss of Fiber-1 with 1 N external force at various positions (polar diagram).Experimental results of the cylindrical array sensor under the 1 N force in longitudinal and circumferential position.

In the experiments, a concentrated force, equal to the force used in simulations, was applied at different longitudinal and circumferential points on the flexible shell to investigate the light intensity modulation in Fiber-1. Figure 10a representatively shows variation in the light intensity measured by the sensor to the variation in the external force at various frequencies, i.e., 0.5, 1, 1.5 Hz, located at (2 mm, 0°). The results showed that the light intensity for this load case decreased by 24% synchronously with the external force. In addition, the light intensity loss decreased 18% ± 5% for a 2 mm change in the longitudinal position *d* of the external force at ϕ=0°. A similar range of intensity loss has been observed in the validation tests on our previous VBR-based sensor reported in [31,37]. Additionally, the post-processing showed that. on average, a minimum of 0.012 ± 0.004 N was required to incur an intensity loss of 1% in the optical fiber’s output intensity with external force at (2 mm, 0°). Moreover, the difference between the theoretical intensity loss percentage (simulation) and experimental observation was 11% ± 3% for 1N external force at various positions (Figure 10c). In addition, the sensitivity of the proposed sensor with respect to change in the external force $S_{F}$ and change in the longitudinal position of the force $S_{d}$ was calculated as:

(6) $$S_{F}=\frac{\Delta I\%}{\Delta F}=\frac{24\%}{1N}=24\;\left(\frac{1}{N}\right),$$

(7) $$S_{d}=\frac{\Delta I\%}{\Delta d}=\frac{18\%}{2mm}=9\;\left(\frac{1}{mm}\right),$$

where ΔI% represents the average light intensity loss percentage at the peak external force, i.e., 1N, ΔF and Δd represent the range of the change in external force and step change in the longitudinal position of the external force for which their corresponding ΔI% were measured. Moreover, our preliminary proof-of-concept results reported in [30] confirmed that the intensity in the optical fiber monotonically decreases when increasing the maximum deflection. The same trend was observed in the experimental results in the current study. To compare the trends, the variation in the normalized values of the maximum intensity loss in Fiber-1 in response to 1 N force was compared with its maximum deflection under the same force. The intensity losses were normalized with respect to the maximum observed intensity loss (from the experiment) and the deflection data were normalized with respect to the maximum deflection of Fiber-1 (from the simulation). Figure 10b shows the comparison of the observed normalized intensity loss and maximum deflection of Fiber-1.

In addition, to show the effect of changing the circumferential position of the external force on the intensity loss and comparison with the simulation, Figure 10c shows the polar diagram of the results of experiments and simulation. The experimentally observed trends of changes in the intensity loss were in fair agreement with the simulations. Additionally, the results showed that both the circumferential and longitudinal positions of the external force affect the intensity loss. Furthermore, similar to the simulation results, the maximum intensity loss was observed with force located on top of the corresponding indenter of Fiber-1. Figure 7a and Figure 10c show a fair agreement between the observed trends in simulation and experiments. More specifically, Table 4 summarizes the observed variations in the intensity loss for 1 N force at various locations on the shell. Given the fact that the aortic annulus has a circular shape (or oval shape in pathologic conditions), the leaflets of the annulus will completely surround the proposed sensor and push on the shell, similar to a ring.

Therefore, the determination of the longitudinal position of the external force will be of higher clinical importance. In such a configuration, all the fibers will exhibit levels of intensity loss. With proper non-linear calibration, i.e., learning-based rate-dependent methods [38], a mapping between states of the intensity loss in the four fibers and the position and magnitude of the external force can be obtained. Since in this experiment only one fiber was utilized, obtaining such calibration was not possible. However, the feasibility of this form of calibration has previously been shown by the authors in [31,36,37]. Post-processing showed that, as predicted from the simulation, the maximum intensity loss was observed with the external force on top of Indenter-1, i.e., d=2 and ϕ=0. In addition, the intensity loss decreased when increasing the distance of external force with the indenter both longitudinally and circumferentially.

The numerical simulation approach was selected since the mechanical model involved complex geometry, material and geometric non-linearities (e.g., for the flexible shell) and contact mechanics, for which an analytical solution was not available. The results of the simulation showed that the deflection of each fiber (thus, its light intensity modulation) is sensitive to both the circumferential and longitudinal position of the external force. Moreover, a similar trend of intensity loss was observed in experiments with one fiber, i.e., Fiber-1.

Another finding was that as the external force moves away from the fiber in a circumferential direction, its ability to deform the optical fiber (and causing the intensity loss) decreases more rapidly than when it moves along the sensor’s longitudinal direction. Nevertheless, the existence of a 30° overlap between the consecutive indenters allows for capturing the effects of force with the next optical fiber in circumferential order as the external force is lessened on each fiber. In addition, the results of the performed tests verified the capability of the sensor to perform under dynamic loading conditions similar to the use case in TAVI. Additionally, the proposed structure of the substrate allows for passing a separate optical fiber to be coupled with a flexible tip at the end of the sensor which can be used for normal tip force measurement, e.g., as proposed in [27]. This way, the sensor can be used as a miniaturized sensor with the ability to measure three spatial components of the tip forces as well as magnitude, longitudinal and circumferential positions of a lateral force (6 DoF sensor).

## 6. Conclusions

This study was a proof-of-concept to show the feasibility of an array force sensor based on the variable bending radius light intensity modulation principle suitable for use in TAVI procedures. The novelty of the proposed sensor was its potential capability to identify the lateral external force and its location with respect to the sensor. Thanks to the novel design and miniature structure, the proposed sensor can be integrated at the tip of commercially available cardiac catheters used in TAVI, either as an integral part or as an add-on feature.

Moreover, the proposed shell substrate design allows for proper isolation of the sensor to avoid blood penetration inside the shell. In addition, since the proposed design does not employ ferromagnetic or electric components, it is MRI compatible and electrically safe, which are of high usability importance. Another contribution of this study was utilizing the SLA 3D printing technique to fabricate flexible and rigid components with high spatial resolution. This technique facilitates fabricating delicate and complex structural components, should an optimized design (such as provided in [31]) be required. In addition, various providers, including Formlabs Inc., provide a wide range of biocompatible resins for SLA 3D printing which can be exploited to fabricate fully biocompatible sensors. The proposed structure in this study is also adaptable with other sensing principles such as wavelength or phase modulation principles. For such adaptation, the optical fibers used in this sensor should be replaced with FBG or FPI fibers.

## Figures and Tables

**Table 1:** Geometric dimensions and mechanical properties of the sensor used in the finite element study.

	Dimensions	Value	Mechanical Properties	Value
	Outer diameter	6 mm	Hyperelastic	$C_{01}=-24.49$
Flexible shell	Length	10 mm	Two-term Mooney–Rivlin [31]	$C_{10}=25.82$
	Thickness	0.5 mm		
Rigid Substrate	Tip diameter	6 mm	Elastic modulus	0.5 GPa
	Shaft diameter	2 mm	Poisson’s ratio	0.43
Optical fiber	Outer diameter	0.250 mm	Elastic modulus	16.5 GPa
	Free length	8 mm	Poisson’s ratio	0.2

**Table 2:** Simulated geometric parameters and values used in finite element simulation.

Parameter	Values
*d*	$\left(\begin{array}{cccc} 2 & 4 & 6 & 8 \end{array}\right)$ mm
ϕ	from −180° to 180° with 20° step-size

**Table 3:** Maximum deflection of optical fibers when force is applied exactly on top of their corresponding indenters.

Fiber No.	1	2	3	4
Maximum deflection (mm)	0.0788	0.0788	0.136	0.136

**Table 4:** Normalized intensity loss (as a percentage of the maximum) in Fiber-1 with 1 N external force.

ϕ	−60°	−40°	−20°	0°	20°	40°	60°
*d* = 2 mm	3 ± 3	12 ± 4	71 ± 5	100 (Maximum)	74 ± 4	10 ± 5	0 ± 5
*d* = 4 mm	2 ± 4	11 ± 5	51 ± 3	72 ± 6	48 ± 6	7 ± 5	0 ± 4
*d* = 6 mm	2 ± 6	6 ± 4	43 ± 5	61 ± 7	45 ± 4	6 ± 5	0 ± 3
*d* = 8 mm	1 ± 2	4 ± 3	23 ± 3	40 ± 4	24 ± 4	4 ± 4	0 ± 2

## Data Availability

Not applicable.

## References

[1] Seibold U., Kübler B., Hirzinger G. (2005). Prototype of instrument for minimally invasive surgery with 6-axis force sensing capability. ICRA.

[2] Polygerinos P., Seneviratne L.D., Razavi R., Schaeffter T., Althoefer K. (2013). Triaxial catheter-tip force sensor for MRI-guided cardiac procedures. IEEE/ASME Trans. Mechatron..

[3] Sauerland S., Jaschinski T., Neugebauer E.A. (2010). Laparoscopic versus open surgery for suspected appendicitis. Cochrane Database Syst. Rev..

[4] Yip M.C., Yuen S.G., Howe R.D. (2010). A robust uniaxial force sensor for minimally invasive surgery. IEEE Trans. Biomed. Eng..

[5] Masson J.B., Kovac J., Schuler G., Ye J., Cheung A., Kapadia S., Tuzcu M.E., Kodali S., Leon M.B., Webb J.G. (2009). Transcatheter aortic valve implantation: Review of the nature, management, and avoidance of procedural complications. JACC Cardiovasc. Interv..

[6] Hooshiar A., Sayadi A., Dargahi J., Najarian S. (2021). Integral-free Spatial Orientation Estimation Method and Wearable Rotation Measurement Device for Robot-assisted Catheter Intervention. IEEE/ASME Trans. Mechatron..

[7] Hooshiar A., Najarian S., Dargahi J. (2020). Haptic Telerobotic Cardiovascular Intervention: A Review of Approaches, Methods, and Future Perspectives. IEEE Rev. Biomed. Eng..

[8] Hooshiar A., Razban M., Bandari N.M., Dargahi J. Sensing principle for real-time characterization of viscoelasticity in the beating myocardial tissue. Proceedings of the 2017 IEEE International Conference on Computational Intelligence and Virtual Environments for Measurement Systems and Applications (CIVEMSA).

[9] Bandari N., Dargahi J., Packirisamy M. (2020). Tactile Sensors for Minimally Invasive Surgery: A Review of the State-of-the-art, Applications, and Perspectives. IEEE Access.

[10] Fontanelli G.A., Buonocore L.R., Ficuciello F., Villani L., Siciliano B. (2020). An External Force Sensing System for Minimally Invasive Robotic Surgery. IEEE/ASME Trans. Mechatron..

[11] Kumar N., Wirekoh J., Saba S., Riviere C.N., Park Y.L. (2020). Soft Miniaturized Actuation and Sensing Units for Dynamic Force Control of Cardiac Ablation Catheters. Soft Robot..

[12] Zhang Y., Ju F., Wei X., Wang D., Wang Y. (2020). A Piezoelectric Tactile Sensor for Tissue Stiffness Detection with Arbitrary Contact Angle. Sensors.

[13] Lee J.i., Lee S., Oh H.M., Cho B.R., Seo K.H., Kim M.Y. (2020). 3D Contact Position Estimation of Image-Based Areal Soft Tactile Sensor with Printed Array Markers and Image Sensors. Sensors.

[14] Mohammadi A., Xu Y., Tan Y., Choong P., Oetomo D. (2019). Magnetic-based soft tactile sensors with deformable continuous force transfer medium for resolving contact locations in robotic grasping and manipulation. Sensors.

[15] Koike R., Sakaino S., Tsuji T. (2019). Hysteresis compensation in force/torque sensors using time series information. Sensors.

[16] Uzun D., Ulgen O., Kocaturk O. (2020). Optical Force Sensor with Enhanced Resolution for MRI Guided Biopsy. IEEE Sens. J..

[17] Ahmadi R., Packirisamy M., Dargahi J. (2012). Innovative optical microsystem for static and dynamic tissue diagnosis in minimally invasive surgical operations. J. Biomed. Opt..

[18] Hooshiar A., Bandari N.M., Dargahi J. Image-based estimation of contact forces on catheters for robot-assisted cardiovascular intervention. Proceedings of the Hamlyn Symposium on Medical Robotics 2018.

[19] Jolaei M., Hooshiar A., Dargahi J., Packirisamy M. (2020). Toward Task Autonomy in Robotic Cardiac Ablation: Learning-based Kinematic Control of Soft Tendon-driven Catheters. Soft Robot..

[20] Jolaei M., Hooshiar A., Sayadi A., Dargahi J., Packirisamy M. Sensor-free Force Control of Tendon-driven Ablation Catheters through Position Control and Contact Modeling. Proceedings of the 2020 42nd Annual International Conference of the IEEE Engineering in Medicine & Biology Society (EMBC).

[21] Hooshiar A., Payami A., Dargahi J., Najarian S. (2021). Magnetostriction-based force feedback for robot-assisted cardiovascular surgery using smart magnetorheological elastomers. Mech. Syst. Signal Process..

[22] Hooshiar A., Alkhalaf A., Dargahi J. (2020). Development and Assessment of a Stiffness Display System for Minimally Invasive Surgery based on Smart Magneto-rheological Elastomers. Mater. Sci. Eng. C.

[23] Alkhalaf A., Hooshiar A., Dargahi J. (2020). Composite magnetorheological elastomers for tactile displays: Enhanced MReffect through Bi-layer composition. Compos. Part B Eng..

[24] Ozioko O., Navaraj W., Hersh M., Dahiya R. (2020). Tacsac: A wearable haptic device with capacitive touch-sensing capability for tactile display. Sensors.

[25] Konstantinova J., Jiang A., Althoefer K., Dasgupta P., Nanayakkara T. (2014). Implementation of tactile sensing for palpation in robot-assisted minimally invasive surgery: A review. IEEE Sens. J..

[26] Polygerinos P., Ataollahi A., Schaeffter T., Razavi R., Seneviratne L.D., Althoefer K. (2011). MRI-compatible intensity-modulated force sensor for cardiac catheterization procedures. IEEE Trans. Biomed. Eng..

[27] Noh Y., Liu H., Sareh S., Chathuranga D., Wurdemann H., Rhode K., Althoefer K. (2016). Image-based optical miniaturized three-axis force sensor for cardiac catheterization. IEEE Sens. J.

[28] Li T., Shi C., Ren H. (2018). Three-dimensional catheter distal force sensing for cardiac ablation based on fiber Bragg grating. IEEE/ASME Trans. Mechatron..

[29] Li T., Pan A., Ren H. (2019). A High-Resolution Tri-axial Catheter-tip Force Sensor with Miniature Flexure and Suspended Optical Fibers. IEEE Trans. Ind. Electron..

[30] Bandari N.M., Hooshiar A., Packirisamy M., Dargahi J. (2016). Optical Fiber Array Sensor for Lateral and Circumferential Force Measurement Suitable for Minimally Invasive Surgery: Design, Modeling and Analysis. Specialty Optical Fibers.

[31] Bandari N., Dargahi J., Packirisamy M. (2020). Miniaturized optical force sensor for minimally invasive surgery with learning-based nonlinear calibration. IEEE Sens. J..

[32] Gauthier R.C., Ross C. (1997). Theoretical and experimental considerations for a single-mode fiber-optic bend-type sensor. Appl. Opt..

[33] Bandari N.M., Hooshair A., Packirisamy M., Dargahi J. (2018). Bending-based formulation of light intensity modulation for miniaturization of optical tactile sensors. Optical Sensors.

[34] Bandari N., Dargahi J., Packirisamy M. Validation of a Variable Bending Radius Sensing Principle for Optical-fiber Tactile Sensors. Proceedings of the 2019 Photonics North (PN).

[35] Spotts M.F., Shoup T.E., Hornberger L.E. (2006). Design of Machine Elelments.

[36] Bandari N., Dargahi J., Packirisamy M. (2020). Image-based Optical-fiber Force Sensor for Minimally Invasive Surgery with ex-vivo Validation. J. Electrochem. Soc..

[37] Bandari N., Dargahi J., Packirisamy M. Camera-Based Optical-Fiber Tactile Sensor for Intraoperative Grasping Force Measurement. Proceedings of the 237th ECS Meeting with the 18th International Meeting on Chemical Sensors (IMCS 2020).

[38] Kakani V., Cui X., Ma M., Kim H. (2021). Vision-Based Tactile Sensor Mechanism for the Estimation of Contact Position and Force Distribution Using Deep Learning. Sensors.

